# The Impact of Obesity on Reproductive Health and Pregnancy Outcomes

**DOI:** 10.7759/cureus.48882

**Published:** 2023-11-16

**Authors:** Akshat Sahu, Sandhya Pajai

**Affiliations:** 1 Department of Obstetrics and Gynaecology, Jawaharlal Nehru Medical College, Datta Meghe Institute of Higher Education and Research (Deemed to Be University), Wardha, IND

**Keywords:** obese, lifestyle, hormones, reproductive health, obesity

## Abstract

Women carry the majority of the burden of our obesogenic surroundings, with a larger prevalence of obesity than males, a greater impact on fertility and treatment success, and increased maternal and perinatal morbidity and death. Obesity and its associated morbidity are now among our most pressing global health concerns. Women are more susceptible to gaining weight, which has reproductive, coronary, and emotional consequences. The current data on the negative consequences of obesity before conception (fertility issues, assisted reproductive treatment, polycystic ovary disease, overweight and obesity preventative measures, and emotional well-being), pregnancy (preventing excess gestational body weight, gestational diabetes, and preeclampsia, as well as labor and newborn health), and following delivery (the lactation process and breastfeeding, postnatal weight retention, and depressive symptoms) health is summarized. in this review. Along with this, underlying factors, consequences, and solutions to the obesity pandemic are investigated, as well as the mechanisms of obesity's effect on women and men, the epigenetic consequences of masculine obesity, its significant effects on reproductive results, and the implications of the loss of weight preceding to pregnancy as well as during pregnancy. This review suggests study methodologies that might assist in guiding attempts to enhance reproductive health and neonatal health in obese or overweight women.

## Introduction and background

Obesity has evolved into a significant concern for society as the incidence is rising. Obese people are more likely to get cardiovascular disease, neurological deficit, osteoarthritis, endocrine disorders, and reproductive issues. Menstrual abnormalities, gestational issues, and infertility owing to anovulation affect females, whereas reduced testosterone and sperm count affect males. Obese women, in particular, have lower levels of both gonadotropins. Obese men have reduced testosterone followed by decreased luteinizing hormone (LH). The results obtained point to central hypothalamic-pituitary-gonadal (HPG) axis malfunction, particularly on the scale of gonadotropin-releasing hormone (GnRH) neurological function, which is the last brain output for reproductive modulation. Obesity is a metabolic condition characterized by high insulin levels, high cholesterol levels, high leptin levels, and chronic inflammation. For proper pubertal growth and adult reproductive functions, integrated metabolic stimulation with the HPG axis is essential. The last brain stimulus that impacts reproduction is GnRH from the hypothalamus [1,2].

Obesity prevalence has progressively climbed worldwide over the last 30 years [3]. According to the National Family Health Survey-5 data, 23% of females and 22.1% of males are overweight according to the body mass index (BMI) criteria. Based on early findings on the country's abdominal obesity situation, 40% of females and 12% of males have abdominal obesity. Obesity rates in children and young people have risen disproportionately, which may have long-term repercussions in a variety of homeostatic systems, especially reproductive capabilities [4]. According to experts from Oxford University, maternal obesity may pose a lesser obstetric risk than previously considered. Their findings imply that obese moms with a BMI higher than 30 kg/m^2^ might not be required to deliver a child in a hospital [5]. According to the International Federation of Gynecology and Obstetrics, it is reported that mothers' and newborns' health risks are expanding. Obese females should try to shed weight before conceiving to reduce the chances of preterm birth, anomalies in fetal development, significant for gestational age babies, and neonatal mortality [6]. The ideal time to control maternal obesity is before conception. Obese women are more vulnerable to spontaneous abortion. Being obese provides an oxidizing physical environment, which causes stimuli that cause mitochondrial damage, lower the quality of the egg, and interfere with the proper development of the embryo [7].

## Review

Methodology

**Figure 1:**
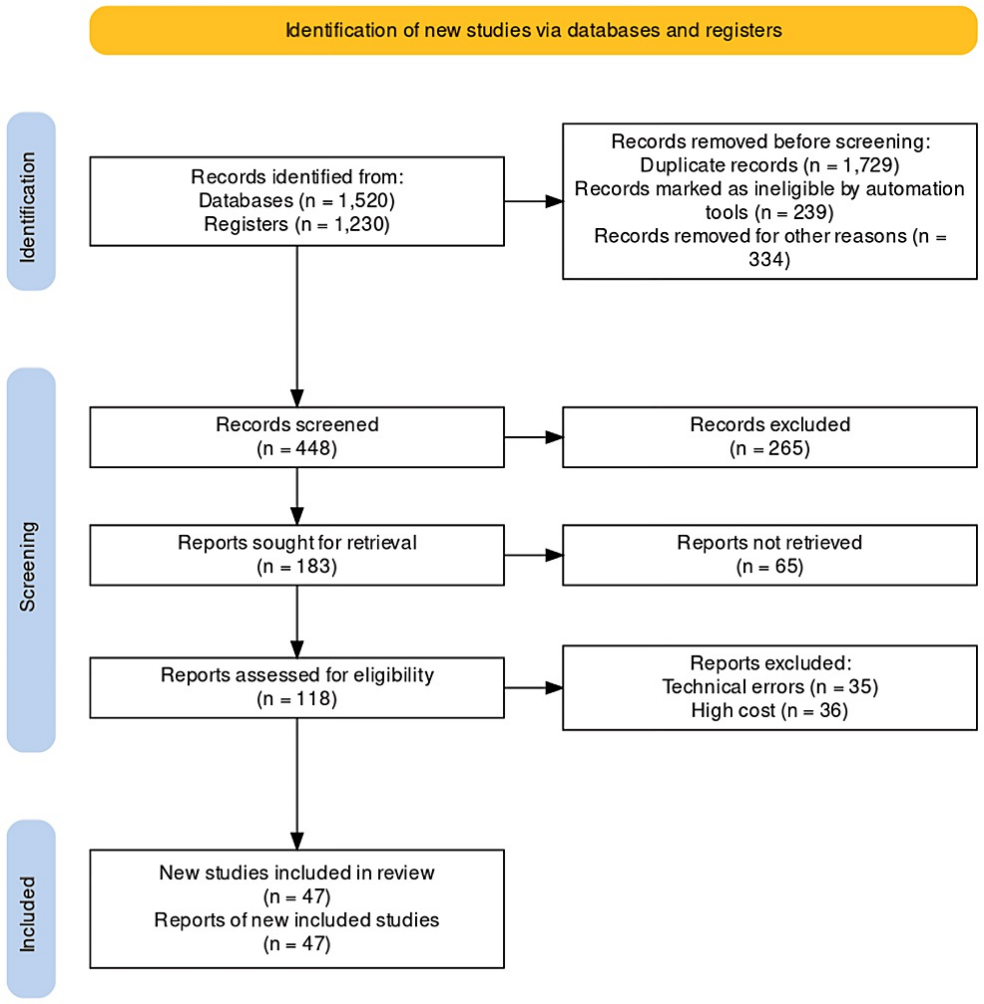
PRISMA flow diagram Reference: Haddaway NR, Page MJ, Pritchard CC, McGuinness LA: PRISMA2020: an R package and Shiny app for producing PRISMA 2020-compliant flow diagrams, with interactivity for optimised digital transparency and open synthesis. Campbell Syst Rev. 2022, 18: e1230. https://doi.org/10.1002/cl2.1230

We searched terms like "obesity and pregnancy", "reproductive health", "fetal development", and "obesity in males" in databases like PubMed. The latest articles were selected for reference. Only review articles were taken into account. For inclusion criteria, we considered articles with the above keywords from the year 2000 to 2023 in the English language. We are excluding studies that were in languages other than English. Studies including subjects of non-reproductive groups were not considered. We also excluded studies whose full-text articles were unavailable. The publications that showed up from these computerized searches, as well as pertinent references in the bibliography for those research, were assessed (Figure 1).

Obesity and female reproductive health

Obesity disrupts the hypothalamic-pituitary-ovarian axis, resulting in shorter luteal phases and reduced amounts of progesterone, LH, and follicle-stimulating hormone in overweight women [8]. Research of over 45,000 transplantation of artificial reproductive embryos found that a greater BMI was associated with a lesser possibility of conceiving successfully when autologous oocytes were utilized, in contrast to when oocytes from healthier and fitter donors were utilized, implying that obesity has an immediate influence on oocytes [9]. Obesity is also linked to alterations in the granulosa cells of the ovary and the fluid in the follicle around the oocyte. Variations in follicular fluid parameters such as insulin, triglycerides, free fatty acids, proinflammatory cytokines, oxidized low-density lipoprotein, and fatty acid composition have been noted in overweight females, implying that oocyte development abnormalities are caused by a variety of mechanisms. It has been observed that stimulating with oxidized low-density lipoprotein produces autophagy as an alternative type of cellular death in granulosa cell cultures as well as apoptosis in endothelial cells. Oxidized low-density lipoprotein can cause oxidative stress and inflammatory response, perhaps leading to anovulation and infertility among obese women and impairing in vitro fertilization (IVF) results [10]. Data from females who had IVF with an oocyte from a donor were used to identify the involvement of the endometrium, although the outcomes were inconclusive. A comprehensive review utilizing information from six different entities found no difference in gestation, implantation of embryos, or spontaneous abortion rates between obese and non-obese females [11]. According to the latest research on pregnancies resulting in surrogacy, raising the surrogate woman's BMI had no relevant influence on IVF, implantation of embryos, or newborn health [12]. Further research on 9587 females demonstrated that the recipient's weight had a detrimental influence on implantation, pregnancy fulfillment, and successful live deliveries, implying that being overweight substantially changes endometrial gene transcription in females throughout the period known as the luteal phase [13]. Therefore, the information indicates that obesity-associated uterine and ovarian changes both play a role in reproductive issues.

Obesity is usually related to elevated insulin levels in the blood, with enhanced ovarian androgen production as a result. The aromatization of these androgens to estrogen is induced by abundant adipose tissue, causing a negative feedback mechanism on the hypothalamic-pituitary-ovarian axis and altering the synthesis of gonadotropin [14]. These changes are the cause of ovulatory dysfunction and menstrual irregularities. Hyperinsulinemia is a crucial player in the pathophysiology of the polycystic cystic ovarian syndrome (PCOS), which is highlighted by oligomenorrhoea and hyperandrogegism. Obesity, when present, promotes insulin resistance and complicates PCOS symptoms. Elevated androgen synthesis in PCOS leads to visceral fat deposition, which exacerbates insulin resistance and hyperinsulinemia, aggravating this vicious cycle [15]. A few researches explored the influence of weight reduction on obese people with PCOS. During a clinical experiment, 149 women suffering from obesity and PCOS were randomly assigned to either 16 weeks of oral contraceptive pill (OCP) treatment, weight loss medical treatments (sibutramine or orlistat) combined with a modification in lifestyle, or a synergistic approach of OCPs and modifications to living habits. Following preconception intervention, women had four cycles of standardized induction of ovulation with clomiphene citrate and timed intercourse. Weight loss of more than 6% was observed in both the lifestyle and combined groups, which was related to increased ovulation rate in contrast to the OCP cohort. Even though the trial was not substantially conducted to identify any variations in live birth rates, there seemed to be a tendency to have the advantage of using lifestyle adjustment [16]. It needs to be underlined that in obese females, losing extra weight before pregnancy can minimize pregnancy risks. Additionally, obesity is the major cause of infertility as it is associated with the pathogenesis of deregulated ovarian function, PCOS, tubal infections, endometriosis, uterine polyps, fibroids, etc.

Obesity and male reproductive health

Obesity rates have risen in sync with reports of increased incidence of low-quality sperm and infertility among males. With male infertility accounting for 45%-50% of infertile couples, an increasing amount of convincing proof associating infertility in men to obesity. Thermal effects, high levels of estrogens, hypogonadotropic hypogonadism, sexual disorders, diabetes mellitus, and genetic changes in sperm are some mechanisms through which obesity may impact spermatogenesis. Aside from the obvious consequences of obesity on the male parent, there appears to be proof that adverse impacts in germ cell deoxyribonucleic acid (DNA) can be passed down to kids via genetic and epigenetic alterations [17].

The mechanism of progression of obesity-related male infertility is multifaceted, with numerous potential influencing variables such as hormone disruptions and potentially temporary epigenetic abnormalities. This is crucial to note that the processes described as possible factors of modified spermatogenesis might possess independent or interconnected effects on being fertile. For instance, epigenetic modifications in sperm, which affect the development of the embryo and the well-being of the baby, can be exacerbated by a variety of components, like endocrine changes, the effect of temperature, and mechanisms of gene expression caused by sleep apnea and stress.

Obesity damages male reproductive health by reducing erectile performance and sperm viability. Multiple research studies have found that BMI has an opposite relationship with concentration of sperm [18,19], motility [20], and normal morphology [21]. Obesity and being overweight were reported to be correlated to an increased occurrence of azoospermia and oligozoospermia in an organized study and systematic review [22]. Obesity is additionally related to a corresponding rise in sperm DNA fragmentation. [23]. Obesity is also linked to aberrant sperm chromatin compaction, a large proportion of spermatozoa having lower mitochondrial membrane potential (MMP), and phosphatidylserine (PS) externalization, a suspicious sign of apoptosis [24].

Obesity is correlated with aberrant sperm chromatin compaction, a substantial number of spermatozoa with lower MMP, and PS externalization, which is a prelude to the mortality of cells [25]. Several hypotheses have been proposed to elaborate obesity-triggered sperm destruction, consisting of aberrant levels of reproductive hormone, insulin resistance, modified adipokine synthesis, scrotal temperature elevation, stress from oxidative damage, and persistent systemic inflammatory conditions. Excess abdominal adipose tissue reduces sex hormone-binding globulin, total and free testosterone (T), inhibin B, and increases T conversion to estradiol as a result of enhanced functioning of aromatase [26,27]. Reduced T levels have a negative influence on spermatogenesis because appropriate intratubular T concentrations are required for Sertoli cell attachment to proliferating germ cells [28]. Obesity additionally results in chromosomal alterations, which can be passed down to descendants.

Pre-gestational obesity and development of the newborn

Obesity among mothers promotes a cascade of metabolic abnormalities that are transmitted down from mother to baby during gestation, with adverse consequences for child and adult well-being. Babies born to obese women are two times more likely to acquire childhood obesity [29]. This is particularly applicable for individuals who were born macrosomic or large for gestational age (>4 kg) [30]. Obese female infants are at high risk of developing hyperinsulinemia and hypoglycemia during the newborn period, which may be attributable in part to gestational diabetes development among several overweight mothers as well [31]. According to some animal studies, maternal obesogenic diets produce insulin resistance and boost fetal blood sugar levels, leading to more rapid pancreatic-cell development and serious glucose intolerance in the child [32]. Long-term research has indicated that babies born to overweight females are highly vulnerable to acquiring neuropsychiatric and mood problems, as well as cognitive deficits. Thus, in-utero exposure to mother obesity prepares the baby for metabolic and neurological disorders in adulthood, and current research indicates that the function of the placenta is crucial in connecting the uterine environment to long-lasting concerns about health [33].

Animal experimental examples of obesity induced by a diet high in fat content show that babies have cognitive deficits, anxiety, and depressive symptoms, as well as social limitations and hyperactivity. In a comparable manner, enhanced cell division was found in the embryonic hypothalamus due to excess amounts of interleukin-6 in vivo and in vitro [34]. Elevated maternal TNF-alpha has been linked to obesity, preterm delivery, and hyperlipidemia, while elevated TNF-alpha from preterm kids' cord blood has been linked to cognitive deficiencies at five years of age [35]. Surprisingly, 35% of children who have autism are additionally vulnerable to childhood obesity, suggesting that the in-utero environment contributes to being prone to both neurodevelopmental and metabolic diseases [36]. Children of obese females might develop behavioral and cognitive abnormalities due to changes in the serotonergic system and hypothalamic-pituitary-adrenal axis caused by elevated proinflammatory cytokines and high-fat diets [37].

A variety of theories are presented to shed light on the apparent relationship connecting pre-pregnancy obesity and neurodevelopmental consequences. As per one prevalent hypothesis, the inflammatory environment that comes with being fat before and throughout pregnancy causes a chain reaction of events that influence brain development and later neurodevelopmental outcomes [38,39]. Preclinical studies corroborate this concept, demonstrating that prenatal obesity caused by a diet comprising of high-fat content causes alterations in inflammatory signaling that affect child microglial functioning, brain development, and future neurobehavioral characteristics [33]. Other mechanisms that may occur include metabolic hormone-induced alterations, such as elevated leptin or insulin circulating levels, or the disruption of serotoninergic activities by interfering with the proper development of the serotonin system. Maternal obesity before pregnancy may potentially be a sign of dietary disparities. For example, a high-fat diet during gestation has been associated with improper function of dopamine and erratic behavior [40].

Whilst preliminary research has been beneficial in understanding some of the probable causes, human population studies have been scarce in number. For instance, methodological problems in human research make disentangling the effects of mother food and maternal pre-pregnancy weight on a child's neurological development difficult. However, initial research suggests a relationship between abnormal gestational inflammatory cytokine levels and children's intelligence quotient and atrial septal defect risk. A significant next step might be linking prenatal maternal obesity exposure to kid cognitive ability via inflammatory signaling [35,41].

Obesity while pregnant does not only pose a risk to the pregnancy's result. An increasing amount of research implies that maternal obesity is responsible for determining the developing fetus's long-term health and wellness. Children affected by maternal obesity and gestational diabetes in the womb have a higher probability of being obese as adults, acquiring type 2 diabetes, and dying from cardiovascular disease. The simultaneous presence of high maternal circulating glucose and high fetal insulin concentrations results in substantial fat accumulation. Inadequate maternal glucose consumption results in increased glucose crossing the placenta and entering the fetal circulation. Research findings corroborate the repercussions of obesity in mothers and gestational diabetes on the health of children, which are conveyed at least in part by alterations in epigenetic events, like alterations in DNA methylation, for example, and perhaps alterations in the gut microbiome [42].

A meta-analysis found that rising maternal BMI significantly higher the probability of child obesity; this association was most convincing with mothers becoming overweight, which increased the likelihood of childhood obesity by 264%, and afterward with maternal overweight, which increased the chances by 89%. Obesity is the result of an intricate blend of physiological, environmental, psychological, social, and behavioral factors [43]. Socioeconomic status, food manufacturing and distribution, lack of access to food, and obesogenic conditions, that foster unhealthy behaviors to which certain people are predisposed through genetics, are further life cycle exposures. Since mothers had access to these complicated circumstances that led to their obesity development, their children will very certainly be subjected to the same complex circumstances that worsen in-utero development and obesity risk [44].

 Interventions and management

Health promotion initiatives aimed at adults and children over the age of 18 are sometimes limited by a shortage of participation from the target population. Though some critical messages regarding diet and nutrition can be deeply established in children's and adults' consciousness, accepting such messages can be shallow. Adequacy of knowledge and education serves a significant part, especially since numerous modifications to lifestyle must be undertaken before pregnancy rather than throughout. Knowing does not guarantee a response since making lifestyle modifications is inherently tricky particularly when those modifications are necessary with no incentive of benefiting the developing baby. It was discovered that many of the women who did so were either unaware of the dangers or stayed in environments where nutritious habits were not standard procedure. The desire to continue their typical social behaviors exceeded the desire to modify their lifestyle. The exact reasons are likely to apply to other hazardous actions before and during pregnancy [45]. While there are defined standards on weight increase for pregnancy in certain countries, and weight monitoring is part of standard prenatal care, the strategy of coping with obesity loses its ability to act. Women's recommendation on what to eat and how much physical exercise to sustain throughout pregnancy is highly generic and frequently misunderstood [46].

There should be no variation in strategy between pregnant and nonpregnant persons in terms of weight management, except that the objective of gestation is to enable weight gain between acceptable ranges instead of to accomplish weight reduction. During pregnancy, women should gain between 11 kg and 16 kg. Ideally, no more than 1 kg to 2 kg of weight increase in the first trimester, followed by 410 g each week after that. Being physically active is crucial, plus formerly sedentary women should sit less, move more, and engage in continuous exercise for no less than fifteen minutes per day, such as brisk walking or going swimming three times per week. Women can reap advantages from the advice of a nutritionist. Expecting women are becoming capable of employing eHealth tools, such as smartphone apps, that are targeted to their weight status [47]. It is an exciting approach to utilizing the teachable moment in pregnancy, with the goal of empowering women to take responsibility for their own health and fitness without being exposed to professional judgment.

## Conclusions

As the global incidence of obesity rises year over year, the potential risk to the well-being and health of pregnant women and their babies, as well as the expense of addressing unfavorable results of pregnancy, becomes more serious. Some approaches can be used to reduce the likelihood of adverse results; however, investment in both health professional training and the execution of therapies customized to the needs of individual women would be necessary for them to be effective in primary care. Weight management conversations should take place during the initial phase of pregnancy, which, while difficult, turns out to be the most significant time to make use of the educational opportunities that the beginning of pregnancy presents. Future prenatal weight reduction requirements in primary care could possibly be most effectively addressed by eHealth strategies for maximum impact.

## References

[1] Lainez NM, Coss D (2019). Obesity, neuroinflammation, and reproductive function. Endocrinology.

[2] Moenter SM (2018). GnRH neurons on LSD: a year of rejecting hypotheses that may have made Karl Popper proud. Endocrinology.

[3] NCD Risk Factor Collaboration (NCD-RisC) (2016). Trends in adult body-mass index in 200 countries from 1975 to 2014: a pooled analysis of 1698 population-based measurement studies with 19·2 million participants. Lancet.

[4] Chavarro JE, Ehrlich S, Colaci DS, Wright DL, Toth TL, Petrozza JC, Hauser R (2012). Body mass index and short-term weight change in relation to treatment outcomes in women undergoing assisted reproduction. Fertil Steril.

[5] Hollowell J, Pillas D, Rowe R, Linsell L, Knight M, Brocklehurst P (2014). The impact of maternal obesity on intrapartum outcomes in otherwise low risk women: secondary analysis of the Birthplace national prospective cohort study. BJOG.

[6] Hanson MA, Bardsley A, De-Regil LM (2015). The International Federation of Gynecology and Obstetrics (FIGO) recommendations on adolescent, preconception, and maternal nutrition: “Think nutrition first”. Int J Gynaecol Obstet.

[7] Mariona FG (2016). Perspectives in obesity and pregnancy. Womens Health (Lond).

[8] Santoro N, Lasley B, McConnell D (2004). Body size and ethnicity are associated with menstrual cycle alterations in women in the early menopausal transition: the study of Women’s Health Across the Nation (SWAN) daily hormone study. J Clin Endocrinol Metab.

[9] Luke B, Brown MB, Stern JE, Missmer SA, Fujimoto VY, Leach R (2011). Female obesity adversely affects assisted reproductive technology (ART) pregnancy and live birth rates. Hum Reprod.

[10] Bausenwein J, Serke H, Eberle K (2010). Elevated levels of oxidized low-density lipoprotein and of catalase activity in follicular fluid of obese women. Mol Hum Reprod.

[11] Jungheim ES, Macones GA, Odem RR, Patterson BW, Lanzendorf SE, Ratts VS, Moley KH (2011). Associations between free fatty acids, cumulus oocyte complex morphology and ovarian function during in vitro fertilization. Fertil Steril.

[12] Coyne K, Whigham LD, O'Leary K, Yaklic JK, Maxwell RA, Lindheim SR (2016). Gestational carrier BMI and reproductive, fetal and neonatal outcomes: are the risks the same with increasing obesity?. Int J Obes (Lond).

[13] Bellver J, Martínez-Conejero JA, Labarta E (2011). Endometrial gene expression in the window of implantation is altered in obese women especially in association with polycystic ovary syndrome. Fertil Steril.

[14] Jungheim ES, Moley KH (2010). Current knowledge of obesity's effects in the pre- and periconceptional periods and avenues for future research. Am J Obstet Gynecol.

[15] Broughton DE, Moley KH (2017). Obesity and female infertility: potential mediators of obesity's impact. Fertil Steril.

[16] Kort JD, Winget C, Kim SH, Lathi RB (2014). A retrospective cohort study to evaluate the impact of meaningful weight loss on fertility outcomes in an overweight population with infertility. Fertil Steril.

[17] Craig JR, Jenkins TG, Carrell DT, Hotaling JM (2017). Obesity, male infertility, and the sperm epigenome. Fertil Steril.

[18] Jensen TK, Andersson AM, Jørgensen N, Andersen AG, Carlsen E, Petersen JH, Skakkebaek NE (2004). Body mass index in relation to semen quality and reproductive hormones among 1,558 Danish men. Fertil Steril.

[19] Hammoud AO, Wilde N, Gibson M, Parks A, Carrell DT, Meikle AW (2008). Male obesity and alteration in sperm parameters. Fertil Steril.

[20] Martini AC, Tissera A, Estofán D, Molina RI, Mangeaud A, de Cuneo MF, Ruiz RD (2010). Overweight and seminal quality: a study of 794 patients. Fertil Steril.

[21] La Vignera S, Condorelli RA, Vicari E, Calogero AE (2012). Negative effect of increased body weight on sperm conventional and nonconventional flow cytometric sperm parameters. J Androl.

[22] Sermondade N, Faure C, Fezeu L (2013). BMI in relation to sperm count: an updated systematic review and collaborative meta-analysis. Hum Reprod Update.

[23] Kort HI, Massey JB, Elsner CW, Mitchell-Leef D, Shapiro DB, Witt MA, Roudebush WE (2006). Impact of body mass index values on sperm quantity and quality. J Androl.

[24] Palmer NO, Bakos HW, Fullston T, Lane M (2012). Impact of obesity on male fertility, sperm function and molecular composition. Spermatogenesis.

[25] Barbagallo F, Condorelli RA, Mongioì LM (2021). Molecular mechanisms underlying the relationship between obesity and male infertility. Metabolites.

[26] Chavarro JE, Toth TL, Wright DL, Meeker JD, Hauser R (2010). Body mass index in relation to semen quality, sperm DNA integrity, and serum reproductive hormone levels among men attending an infertility clinic. Fertil Steril.

[27] Ramlau-Hansen CH, Hansen M, Jensen CR, Olsen J, Bonde JP, Thulstrup AM (2010). Semen quality and reproductive hormones according to birthweight and body mass index in childhood and adult life: two decades of follow-up. Fertil Steril.

[28] Soubry A, Guo L, Huang Z, Hoyo C, Romanus S, Price T, Murphy SK (2016). Obesity-related DNA methylation at imprinted genes in human sperm: results from the TIEGER study. Clin Epigenet.

[29] Zhang S, Rattanatray L, Morrison JL, Nicholas LM, Lie S, McMillen IC (2011). Maternal obesity and the early origins of childhood obesity: weighing up the benefits and costs of maternal weight loss in the periconceptional period for the offspring. Exp Diabetes Res.

[30] Boney CM, Verma A, Tucker R, Vohr BR (2005). Metabolic syndrome in childhood: association with birth weight, maternal obesity, and gestational diabetes mellitus. Pediatrics.

[31] Desoye G, van Poppel M (2015). The feto-placental dialogue and diabesity. Best Pract Res Clin Obstet Gynaecol.

[32] Ford SP, Zhang L, Zhu M (2009). Maternal obesity accelerates fetal pancreatic beta-cell but not alpha-cell development in sheep: prenatal consequences. Am J Physiol Regul Integr Comp Physiol.

[33] Bolton JL, Bilbo SD (2014). Developmental programming of brain and behavior by perinatal diet: focus on inflammatory mechanisms. Dialogues Clin Neurosci.

[34] Sullivan EL, Riper KM, Lockard R, Valleau JC (2015). Maternal high-fat diet programming of the neuroendocrine system and behavior. Horm Behav.

[35] von Ehrenstein OS, Neta GI, Andrews W, Goldenberg R, Goepfert A, Zhang J (2012). Child intellectual development in relation to cytokine levels in umbilical cord blood. Am J Epidemiol.

[36] Granich J, Lin A, Hunt A, Wray J, Dass A, Whitehouse AJ (2016). Obesity and associated factors in youth with an autism spectrum disorder. Autism.

[37] Kim DW, Glendining KA, Grattan DR, Jasoni CL (2016). Maternal obesity leads to increased proliferation and numbers of astrocytes in the developing fetal and neonatal mouse hypothalamus. Int J Dev Neurosci.

[38] Buss C, Entringer S, Davis EP, Hobel CJ, Swanson JM, Wadhwa PD, Sandman CA (2012). Impaired executive function mediates the association between maternal pre-pregnancy body mass index and child ADHD symptoms. PLoS One.

[39] Bilbo SD, Tsang V (2010). Enduring consequences of maternal obesity for brain inflammation and behavior of offspring. FASEB J.

[40] Grissom NM, Herdt CT, Desilets J, Lidsky-Everson J, Reyes TM (2015). Dissociable deficits of executive function caused by gestational adversity are linked to specific transcriptional changes in the prefrontal cortex. Neuropsychopharmacology.

[41] Jones KL, Croen LA, Yoshida CK (2017). Autism with intellectual disability is associated with increased levels of maternal cytokines and chemokines during gestation. Mol Psychiatry.

[42] Godfrey KM, Reynolds RM, Prescott SL, Nyirenda M, Jaddoe VW, Eriksson JG, Broekman BF (2017). Influence of maternal obesity on the long-term health of offspring. Lancet Diabetes Endocrinol.

[43] Heslehurst N, Vieira R, Akhter Z (2019). The association between maternal body mass index and child obesity: a systematic review and meta-analysis. PLoS Med.

[44] Bush NR, Allison AL, Miller AL, Deardorff J, Adler NE, Boyce WT (2017). Socioeconomic disparities in childhood obesity risk: association with an oxytocin receptor polymorphism. JAMA Pediatr.

[45] Meurk CS, Broom A, Adams J, Hall W, Lucke J (2014). Factors influencing women's decisions to drink alcohol during pregnancy: findings of a qualitative study with implications for health communication. BMC Pregnancy Childbirth.

[46] Swift JA, Langley-Evans SC, Pearce J (2017). Antenatal weight management: diet, physical activity, and gestational weight gain in early pregnancy. Midwifery.

[47] Langley-Evans SC, Pearce J, Ellis S (2022). Overweight, obesity and excessive weight gain in pregnancy as risk factors for adverse pregnancy outcomes: a narrative review. J Hum Nutr Diet.

